# Cyanine Dyes for Photo-Thermal Therapy: A Comparison of Synthetic Liposomes and Natural Erythrocyte-Based Carriers

**DOI:** 10.3390/ijms22136914

**Published:** 2021-06-27

**Authors:** Giulia Della Pelle, Andrea Delgado López, Marina Salord Fiol, Nina Kostevšek

**Affiliations:** 1 Department for Nanostructured Materials, Jožef Stefan Institute, 1000 Ljubljana, Slovenia; 2 Jožef Stefan International Postgraduate School, 1000 Ljubljana, Slovenia; 3 Faculty of Pharmacy and Food Science, University of Barcelona, 08028 Barcelona, Spain; andrea.delgadolopez98@gmail.com (A.D.L.); marina1325@hotmail.es (M.S.F.)

**Keywords:** indocyanine green, IR-820, liposomes, erythrocyte membrane vesicles, photo-thermal therapy

## Abstract

Cyanine fluorescent dyes are attractive diagnostic or therapeutic agents due to their excellent optical properties. However, in free form, their use in biological applications is limited due to the short circulation time, instability, and toxicity. Therefore, their encapsulation into nano-carriers might help overcome the above-mentioned issues. In addition to indocyanine green (ICG), which is clinically approved and therefore the most widely used fluorescent dye, we tested the structurally similar and cheaper alternative called IR-820. Both dyes were encapsulated into liposomes. However, due to the synthetic origin of liposomes, they can induce an immunogenic response. To address this challenge, we proposed to use erythrocyte membrane vesicles (EMVs) as “new era” nano-carriers for cyanine dyes. The optical properties of both dyes were investigated in different biological relevant media. Then, the temperature stability and photo-stability of dyes in free form and encapsulated into liposomes and EMVs were evaluated. Nano-carriers efficiently protected dyes from thermal degradation, as well as from photo-induced degradation. Finally, a hemotoxicity study revealed that EMVs seem less hemotoxic dye carriers than clinically approved liposomes. Herein, we showed that EMVs exhibit great potential as nano-carriers for dyes with improved stability and hemocompatibility without losing excellent optical properties.

## 1. Introduction

Cyanine fluorescent dyes are widely used as diagnostic or therapeutic agents. Indocyanine green (ICG) is a tricarbocyanine dye that absorbs and emits in the near-infrared (NIR) region of the spectrum (peak spectral absorption at about 800 nm) and has small absorption in the visible range [1]. The absorbed light is then emitted in the form of fluorescence and heat [2]. Due to its optical properties, ICG is a contrast agent commonly used for in vivo cardiovascular fluorescence imaging and NIR optical imaging for intervention in cases of lymphatic abnormalities [3,4]. It was clinically approved in 1959 by the Food and Drug Administration but was widely used during the Second World War for fluorescence-aided surgery [5,6]. It has been investigated as a photo-thermal agent and photo-sensitizer as well [7]. Not being a good source of singlet oxygen, its possible applications in photodynamic therapy are limited [8]. Despite its excellent optical properties, ICG has some disadvantages for biological applications because of its short plasma residence time (2–4 min), instability in aqueous solutions, and concentration-dependent peak emission location [9]. Despite excellent imaging and clearance properties (ICG follows an almost entirely bile juice route for clearance and has no known metabolites [10]), there are some concerns about its safety in limited applications. The latter is the case of retinal toxicity [11] arising from decreased mitochondrial activity while exposed to a light source, while increasing DNA synthesis. It has been earlier demonstrated that ICG toxicity upon light irradiation is mainly due to the presence of dissolved photo-degradation products in the cell cytoplasm rather than the generation of singlet oxygen [8,12]. 

**Figure 1:**
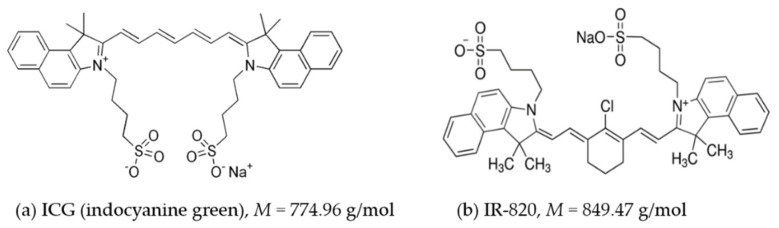
Chemical structure and molecular weight of (**a**) indocyanine green (ICG) and (**b**) IR-820.

A similar cyanine dye, IR-820, has optical and thermal generation properties similar to those of ICG but with higher solubility and improved in vitro and in vivo stability [9,13]. An additional chlorobenzene ring in the IR-820 structure is expected to increase molecular stability [9]. This indicates that IR-820 could be an alternative to ICG in clinical use. The chemical structures of ICG and IR-820 are shown in Figure 1.

To overcome the issues with stability and degradation, encapsulation into nano-carriers, such as liposomes, might help reduce the plasma degradation of ICG, extend the circulation time, and reduce toxicity [3,14]. However, due to the synthetic nature of liposomes, they can be recognized and eliminated as a foreign substance by the immune system [15]. Efforts to extend nanoparticle residence time in vivo have inspired many strategies of particle surface modifications to bypass macrophage uptake and systemic clearance. Using the body’s own cells for the preparation of a safer delivery system is the most recent and advanced approach in the field of nanomedicine and represents an elegant, safe, and effective solution. Disguised with cell membranes, dyes can act as autogenous cells and thus ensure inherent biocompatibility. Among different circulatory cells, erythrocytes are the most abundant and thus can be isolated in sufficiently large quantities to decrease the complexity and cost of the treatment [16]. Erythrocytes have cell markers (CD47) on their surface that provide them with the ability to escape immune system attack [17,18]. For example, a biodistribution study on erythrocyte-coated iron oxide nanoparticles showed a longer circulation time than polyethylene-glycol-coated ones and lower accumulation in the liver and spleen, two major organs of the reticuloendothelial system (RES) [13]. To date, only a few reports can be found on the encapsulation of ICG into erythrocyte-membrane-based carriers, hereinafter referred to as erythrocyte membrane vesicles (EMVs). Bahmani et al. [19] reported the preparation of ICG-loaded EMVs, and high-energy laser irradiation (19.7 W/cm^2^) showed heating due to photo-thermal conversion (investigated only single concentration and single laser power). Interestingly, free ICG heated more than ICG EMVs, which also showed stronger photo-degradation. In later research, they demonstrated diagnostic (fluorescence imaging) and therapeutic capabilities (photo-destruction of SKBR3 breast cancer cells) of ICG-EMVs in vivo [20]. These reports clearly show the high potential of fluorescent-dye-encapsulated EMVs for theranostic purposes (a portmanteau for therapeutic + diagnostic) [21]. However, more research to elucidate the basic physicochemical properties of such nanostructures is warranted, which will help optimize the protocols for the development of safe cell-based nano-carriers with superior theranostic properties compared with free dyes.

Therefore, this article represents the first systematic, comparative study where both types of nano-carriers, EMVs and clinically more established liposomes, loaded with either ICG or IR-820 as an alternative, were compared to free dyes in terms of thermal stability, photo-stability, and hemotoxicity. The aim of this study is to answer the following questions: (a) whether encapsulation into EMVs can improve thermal stability and photo-stability of free dyes, both of which are important to achieve the maximum-possible photo-thermal effect in cancer therapy; (b) how this compares to liposomal formulation; and (c) whether IR-820, as a structurally similar fluorescent dye to ICG, has similar optical properties, stability, and safety and thus can be used as a cheaper alternative to ICG in medical applications.

## 2. Results and Discussion

### 2.1. Light Absorption Properties of ICG and IR-820 in Different Solvents

**Figure 2:**
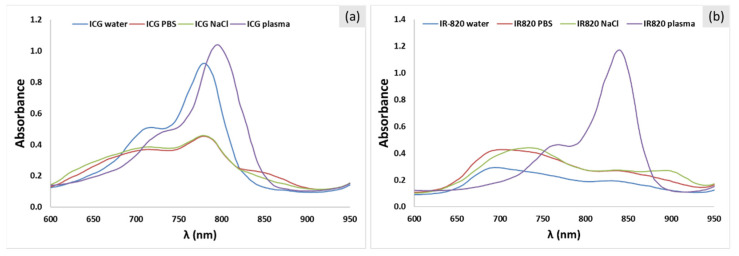
Absorption spectra of (**a**) ICG and (**b**) IR-820 in water, PBS buffer, 0.9% NaCl(aq), and blood plasma. The dye concentration is 10 μg/mL in all cases.

The absorption spectra of ICG solutions in water, PBS buffer, 0.9% NaCl(aq), and blood plasma are shown in Figure 2a. ICG solution in ultrapure water showed two distinctive peaks: ICG monomers (λ = 780 nm) and H-type dimers (λ = 718 nm) [22,23,24]. In this concentration range, the monomeric form is predominant [23,25]. With the addition of salts (NaCl) and consequently increased ionic strength of the media, dimerization becomes more significant [22]. Therefore, saline and PBS solutions have a similar position of absorption peaks than pure water solutions but a significantly lower absorbance value (especially for the monomeric peak) and broader peaks. Additionally, the peak for the J-type dimer (λ > 840 nm) appeared. H-type dimers (λ = 718 nm) absorb at a shorter wavelength than monomers due to side-to-side arrangement of two ICG molecules, and the J-type oligomer absorbs at a longer wavelength than monomers due to the head-to-tail arrangement of ICG molecules [2]. These significant changes in the absorption properties indicate that the selection of solvent plays an important role and can modify the dye’s optical and photo-thermal properties. To test biologically relevant conditions, the absorption spectrum for ICG in blood plasma was recorded as well. Interestingly, when dissolved in plasma, ICG showed higher absorption and red shift of absorption peaks compared to the aqueous solution. The peak for H-type dimers shifted from 718 to 768 nm, and the monomeric peak shifted from 780 to 840 nm. Additionally, the monomeric peak increased compared to the ICG solution in pure water, indicating that plasma proteins stabilize the monomeric form of ICG [23]. ICG therefore is supposed to be adsorbed by plasma proteins, such as albumin, as already demonstrated through human serum albumin titration.

Despite the structural similarity, the absorption spectra of IR-820 solutions (Figure 2b) differed significantly from ICG solutions (Figure 2a). IR-820 in pure water had two absorption peaks at 690 and 813 nm [9]. At the same concentration (10 μg/mL), ICG had significantly higher absorption than IR-820, which could make ICG a more efficient photo-thermal agent [9]. In contrast to ICG, the addition of salts to IR-820 (solution in PBS and saline) led to absorption increase and the occurrence of three less distinctive peaks, indicating the presence of dimerization and the formation of higher oligomers. Interestingly, in the case of 0.9% NaCl(aq), absorption peaks red-shifted compared to IR820 in water. Similar to ICG, the IR-820 solution in plasma had significantly higher absorption than other IR-820 solutions, with two distinctive peaks at 768 and 840 nm. Plasma proteins interact with dyes in a similar manner, making monomers the most stable form. The monomer peak position for ICG and IR-820 in plasma was 794 and 840 nm, respectively.

### 2.2. Temperature-Stability Study of ICG and IR-820 in Different Solvents

**Figure 3:**
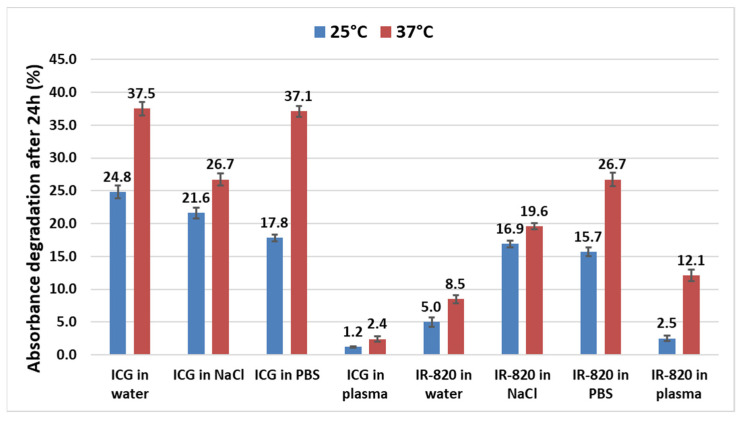
Temperature stability study of ICG and IR-820 solutions in different solvents at 25 and 37 °C. Absorbance decrease (%) after 24 h incubation in the dark was calculated from the absorbance peak values measured before and after incubation. Measurements were performed in triplicate. Statistical analysis confirmed that the means of each data pair (sample at 25 and 37 °C) are not equal, and therefore, the absorbance values differ significantly (*p* < 0.05).

Despite the broad use of ICG for different medical applications, no systematic study on its temperature stability in different biologically relevant solvents can be found. Only stability studies in aqueous solutions have been reported [9,25,26,27]. To fill this gap, we investigated solutions of both dyes (ICG and IR-820) in water, PBS buffer, 0.9% NaCl(aq), and blood plasma. All samples contained 10 μg/mL of the dye and were incubated for 24 h at 4, 25, and 37 °C in the dark. This concentration was chosen since it has been proved that the monomeric form is still predominant without signs of aggregation [25,26]. Single-wavelength absorbance was measured every hour at the absorption maximum determined for each sample from the spectra shown in Figure 2. The calculated percentage absorbance decrease after 24 h incubation at 25 and 37 °C for all tested solutions is presented in Figure 3. At 25 °C, ICG solutions in water, 0.9% NaCl(aq), and PBS showed evidence of degradation, with 24.8%, 21.6%, and 17.8% absorbance decrease, respectively, consistently with literature data [2]. In aqueous solutions, ICG degrades to leucoforms due to the saturation of double bonds. Leucoforms can further fragment into smaller molecules with intact aromatic end groups [26]. Interestingly, the ICG solution in plasma exhibited excellent stability with only 1.2% degradation after 24 h. At higher temperature (37 °C), the degradation rate even increased in all cases, reaching up to 37% in water and PBS. Importantly, the percentage degradation in plasma remained low, indicating possible stabilization of ICG by plasma proteins, avoiding the formation of J or H aggregates [19,24], the latter being responsible, at higher temperatures, for facilitating the exchange of single oxygen radicals known to trigger ICG degradation, as it happens in light-induced degradation [2,8,26].

Interestingly, at both 25 and 37 °C, IR-820 solutions in water, NaCl, and PBS degraded less than ICG solutions, with the highest detected degradation in PBS at 37 °C (26.7%). On the contrary, in plasma, degradation of IR-820 was more pronounced (12.1% at 37 °C) than for ICG (2.4% at 37 °C). From these results, we concluded that IR-820 seems to be more stable in water compared with ICG, while the opposite is valid in plasma, indicating ICG stability superiority in a biologically relevant medium. Stability in NaCl and PBS was slightly in favor of IR-820.

Finally, all solutions were incubated for 24 h at 4 °C in the dark. No drop in absorbance was detected in all cases (graphs not shown), indicating that temperature plays a crucial role in dye degradation, with a higher degradation rate at higher temperature. This has to be considered when planning dye stock solution storage (ideally at 4 °C in the dark) and in vitro and in vivo experiments that are conducted at 37 °C. Fernandez-Fernandez et al. [9] tested the stability of ICG and IR-820 aqueous solutions (0.625 μg/mL) at 4, 22, and 42 °C in the dark and observed a significant fluorescence decrease after 24 h. The most intensive degradation was observed for ICG at 42 °C (>80% signal decrease), and the least degraded sample was IR-820 at 4 and 22 °C (approx. 35% signal decrease). Further, Mindt et al. [27] observed a fluorescence intensity decrease for an ICG aqueous solution (50 μg/mL) of only about 6.3% after 24 h at room temperature and slightly less at 4 °C. Our results, i.e., ICG aqueous solution degradation (10 μg/mL) after 24 h at 25 and 37 °C, fall between the results of these two reports. Deviations can be attributed to the different testing conditions, such as different dye concentrations (higher degradation at lower tested concentration [9]) and measurements of fluorescence [9,27] and, in our case, absorbance.

### 2.3. Photo-Stability Study of ICG and IR-820 Solutions in PBS

**Figure 4:**
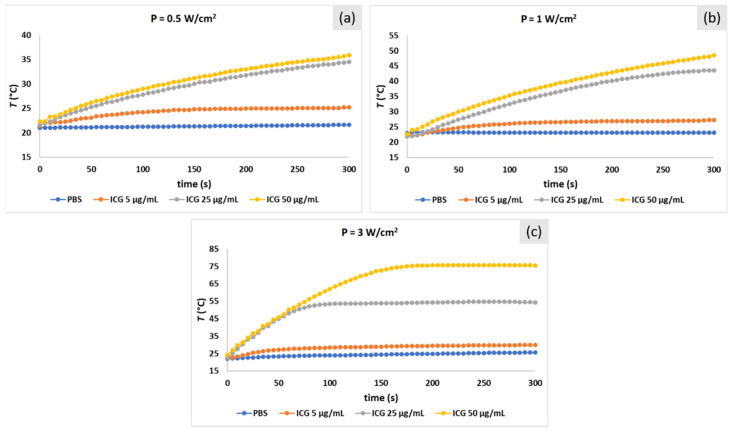
Temperature profiles of ICG solutions (5, 25, and 50 μg/mL) in PBS irradiated with 808 nm laser at laser power (**a**) P = 0.5 W/cm^2^, (**b**) P = 1 W/cm^2^, and (**c**) P = 3 W/cm^2^. PBS solution was irradiated as a control.

To study photo-stability, ICG and IR-820 solutions in PBS with three different concentrations (5, 25, and 50 μg/mL) were irradiated with three different laser powers (P = 0.5, 1, and 3 W/cm^2^). PBS was selected as a biologically relevant medium since most of the tested samples from the literature are dispersed in PBS or similar buffers. Heating profiles for ICG solutions are shown in Figure 4. The calculated absorbance decrease after laser irradiation for all solutions is listed in Table 1. As expected, from the calculated *ΔT* (difference between final and initial temperatures after 5 min irradiation), it is evident that higher laser power and higher ICG concentration lead to higher *ΔT*. At laser power P = 3 W/cm^2^, temperature saturates at 76 and 54 °C for an ICG concentration of 50 and 25 μg/mL, respectively. Saturation is a result of the dye-intensive decomposition at higher light irradiance, which is visible as a color change from green to yellow and detected as an absorption decrease.

**Figure 5:**
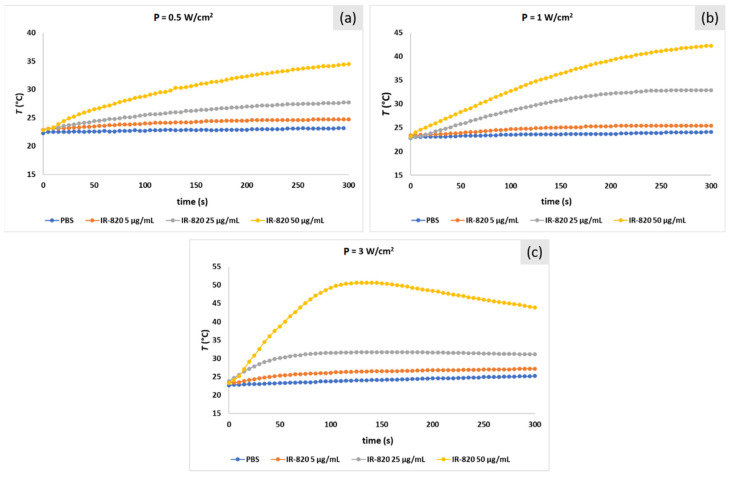
Temperature profiles of IR-820 solutions with three different concentrations (5, 25, and 50 μg/mL) in PBS irradiated with 808 nm laser at laser power (**a**) P = 0.5 W/cm^2^, (**b**) P = 1 W/cm^2^, and (**c**) P = 3 W/cm^2^. PBS solution was irradiated as a control.

Heating profiles of IR-820 solutions are shown in Figure 5. The calculated absorbance decrease after laser irradiation for all solutions is listed in Table 2. Similar to ICG solutions, *ΔT* is a function of laser power and dye concentration. The highest *T* (52 °C) was achieved at the highest laser power P = 3 W/cm^2^ and IR-820 concentration = 50 μg/mL (Figure 5c). Interestingly, due to the dye decomposition, in this extreme case, the temperature did not saturate at the maximum value but slowly decreased.

**Figure 6:**
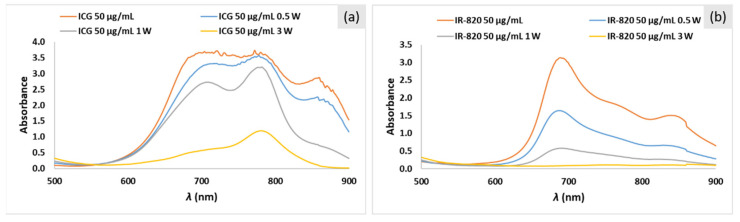
Absorption spectra of (**a**) ICG and (**b**) IR-820 solutions in PBS (50 μg/mL) before and after laser irradiation with P = 0.5, 1, and 3 W/cm^2^ for 5 min.

Absorption spectra before and after laser irradiation for ICG and IR-820 solutions (50 μg/mL) in PBS are shown in Figure 6a and Figure 6b, respectively. Results clearly indicate that higher laser power leads to a stronger absorption decrease after the same irradiation time (5 min). For example, for the 50 μg/mL ICG solution in PBS, absorbance decreased after 5 min of irradiation by 3%, 13%, and 68% at 0.5, 1, and 3 W/cm^2^, respectively (Table 1 and Figure 6a). For the 50 μg/mL IR-820 solution, the percentage decrease was 53%, 81%, and 93% at 0.5, 1, and 3 W/cm^2^, respectively (Table 2 and Figure 6b). The same trend was observed for ICG and IR-820 solutions with concentrations of 5 and 25 μg/mL in PBS but with lower absorbance values (Table 1 and Table 2). In summary, a higher laser power leads to a higher temperature increase but with more severe dye degradation. In agreement with our results, Saxena et al. [26] showed faster ICG degradation in aqueous solutions when exposed to laser light (λ = 786 nm, P = 70 mW) than ambient light. Degradation half-times were calculated to be 2.3 and 14.4 h, respectively. Acceleration in ICG degradation with a stronger light source was attributed to the increase in the production of photo-excited ICG molecules, which tend to form radicals, which further lead to faster degradation toward leucoforms. ICG and IR820 degradation products (Figure 6) did not show absorbance in the same range of parental molecules, thus confirming previously reported data for one decomposition compound analyzed by Engel et al. [8].

In clinical photo-thermal applications, the required temperature increase is in the range of 5 °C (e.g., mild hyperthermia region, 41–43 °C), which can be easily achieved with low laser power to reduce dye degradation and low dye concentration to reduce the risk of toxicity. For example, heating for approx. 5 °C can be observed with 5 μg/mL and P = 1 W/cm^2^ for ICG and 25 μg/mL and P = 0.5 W/cm^2^ for IR-820. As already expected from higher absorbance, under the same conditions, ICG showed a higher photo-thermal effect than IR-820. Importantly, not only heat generation (photo-thermal therapy) but also light-induced degradation products (photo-dynamic therapy) should be carefully considered as an additional toxic event in the exposed cells. Namely, ICG photo-degrades via oxidative C–C cleavage of the polymethine chain, where several different degradation products are formed, depending on the environmental conditions (e.g., oxygen concentration, presence of ^1^O_2_ quenchers) [28]. To enhance dye stability, in our study, ICG and IR-820 were encapsulated into liposomes and erythrocyte membrane vesicles (EMVs).

### 2.4. Preparation of ICG- and IR-820-Loaded Carriers

A comparison, in terms of size and zeta potential, of free carriers (liposomes and EMVs) and loaded ones was carried out, and the encapsulation efficiency (EE) was in parallel estimated. As listed in Table 3, encapsulation of ICG or IR-820, at a working concentration, did not influence liposome size compared to empty liposomes (115 nm). However, the polydispersity index increased, indicating broader size distribution due to uneven dye encapsulation. This could arise from uneven encapsulation among carriers of quantity of dyes, since ICG is known to locate within the double layer and increase the size accordingly [3]. No statistically significant difference in the EE between ICG (45%) and IR-820 (46%) was observed (Table 3). The same parameters were measured for EMVs. Both size and the PDI of EMVs were found to be larger than for liposomes due to the more complex membrane structure (presence of proteins, lipid rafts, and other molecules that contribute to the hydrodynamic diameter) and extrusion process. The EE of IR-820- and ICG-loaded EMVs was determined to be 57% and 53%, respectively. From the comparison of the results obtained for dye-loaded liposomes and EMVs, we can conclude that on average, the EE for both dyes is around 50%.

**Figure 7:**
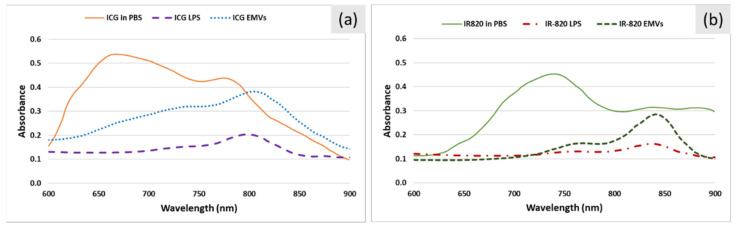
Characterization of purified ICG- and IR-820-loaded liposomes and EMVs. (**a**) Absorption spectra of ICG samples: ICG in PBS, ICG-loaded liposomes (LPS), and ICG-loaded EMVs. (**b**) Absorption spectra of IR-820 samples: IR-820 in PBS, IR-820-loaded liposomes (LPS), and IR-820-loaded EMVs. Liposomes and EMVs are dispersed in PBS buffer.

The absorbance spectra of ICG- and IR-820-loaded carriers are shown in Figure 7a and Figure 7b, respectively. The shape of the spectra for dye-loaded liposomes and EMVs was similar. However, they strongly differed from the spectra of free dyes in PBS. Interestingly, spectra for dye-loaded liposomes and EMVs resembled more the spectra obtained in plasma (Figure 2), indicating stabilization of the monomeric form by lipid components of the carriers.

### 2.5. Temperature Stability of Dye-Loaded Liposomes and EMVs

**Figure 8:**
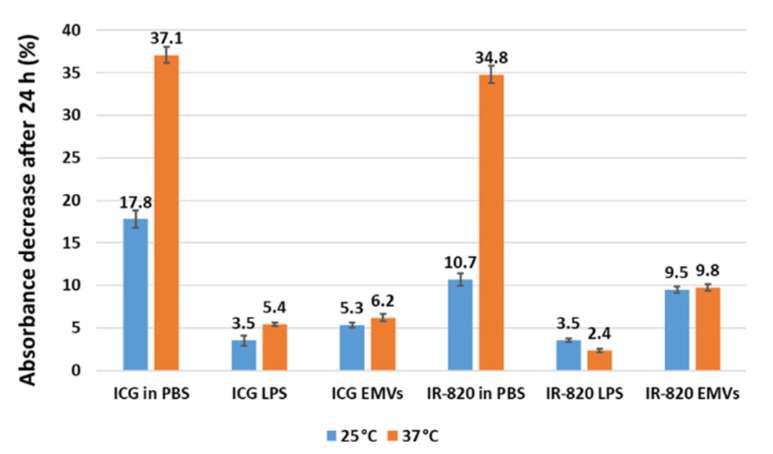
Temperature stability study of ICG- and IR-820-loaded carriers at 25 and 37 °C. Absorbance decrease (%) after 24 h incubation in the dark was calculated from the absorbance peak values measured before and after incubation. Measurements were performed in triplicate. Statistical analysis confirmed that the means of each data pair (sample at 25 and 37 °C) are not equal, and therefore, the absorbance values differ significantly (*p* < 0.05), except for IR-820-loaded EMVs at 25 and 37 °C, where no statistically significant difference could be observed.

To evaluate the temperature stability of dye-loaded carriers, samples were incubated at 25 and 37 °C for 24 h. Interestingly, liposomes and EMVs significantly improved the dye stability compared to the stability of free dyes in PBS, even at higher temperature (37 °C) (Figure 8). The highest detected percentage degradation of ICG in EMVs was 6.2%, which is a 6-fold improvement compared to the free dye in PBS (37%). Similarly, IR-820 in EMVs degraded by about 10% compared to a 3.5-fold higher degradation in PBS at 37 °C. These data confirm for EMVs, too, what has been already observed in the case of polymeric nanostructure encapsulation [29] and PGLA [30]. Importantly, it seems that liposomes and EMVs equally protect dyes from degradation at 25 and 37 °C. The observed percentage degradation was similar to free dyes in plasma but with other added functionalities that nano-carriers can offer, i.e., preparation of theranostic nanostructures and a prolonged circulation time. This protective effect from temperature degradation is due to supposedly reduced vibrational, rotational, and translation motions among ICG molecules, causing less pronounced radical formation and subsequent degradation [2], as observed in the case of plasma (Section 2.2).

### 2.6. Photo-Thermal Effect of ICG- and IR-820-Loaded Liposomes and EMVs

**Figure 9:**
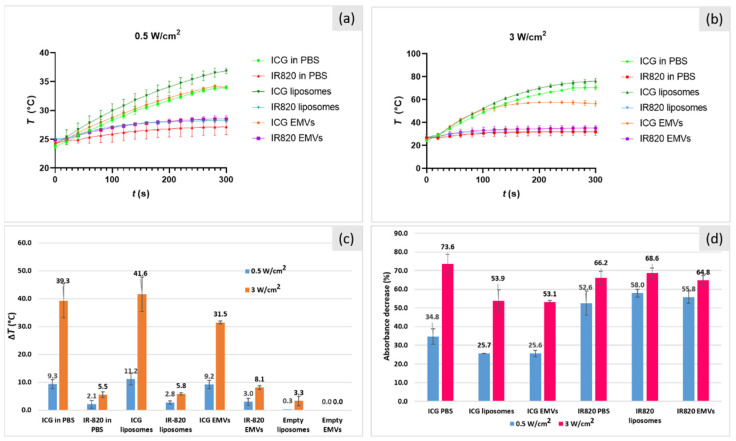
Photo-thermal experiments on dye-loaded nano-carriers. Heating profiles after 5 min irradiation with laser power (**a**) 0.5 and (**b**) 3 W/cm^2^. Data were reported as the mean ± SD. (**c**) Calculated temperature increase after 5 min laser irradiation. An increase in temperature (*ΔT*) was calculated by subtracting the initial temperature (*t* = 0 s) from the maximum temperature reached. Three measurements were performed for each sample. (**d**) Absorbance decrease (%) as an indicator of dye decomposition was calculated from the absorbance peak values measured before and after irradiation. Three measurements were performed for each sample. Observed lower degradation for ICG-loaded carriers, at both laser powers, was proven to be statistically significant with Brown–Forsyth ANOVA (*p* < 0,05). No statistically relevant differences were confirmed for IR820-loaded carriers.

Photo-stability and laser-induced heating capability of dye-loaded nano-carriers was studied by irradiating samples with different laser powers (P = 0.5 and 3 W/cm^2^) for 5 min. Heating profiles for ICG and IR-820 samples are shown in Figure 9a and Figure 9b, respectively. Calculated Δ*T* values obtained after 5 min laser irradiation for all solutions are presented in Figure 9c. Expectedly, a higher laser power led to a higher temperature increase (Δ*T*). Empty carriers showed minimal heating (for liposomes, up to 3.3 °C at P = 3 W/cm^2^). For example, for ICG- and IR-820-loaded liposomes, at laser power P = 3 W/cm^2^, the temperature increased by 41.6 and 5.8 °C, respectively. At a lower laser power, P = 0.5 W/cm^2^, all ICG samples reached a similar Δ*T* (approx. 10 °C). Interestingly, at a higher laser power, ICG-loaded EMVs showed a lower Δ*T* (31.6 °C) than ICG in PBS and ICG-loaded liposomes (39.3 and 41.6 °C, respectively). This is in accordance with the observation of Bahmani et al. [19], where ICG-loaded EMVs exhibited a lower Δ*T* than free ICG. However, they observed strong photo-degradation and a temperature decrease during irradiation, which was not the case in our experiment. Even though experiments were carefully designed to have the same dye concentration in all cases (10 μg/mL), this discrepancy for ICG-loaded EMVs at P = 3 W/cm^2^ might be attributed to a small deviation in the actual ICG concentration due to extrusion processes, which we avoided to use in this experimental design. On the contrary, IR-820-loaded EMVs showed a similar heating effect to IR-820-loaded liposomes at both laser powers (and both nano-carriers exhibited a similar Δ*T* than free IR-820). These results indicate that the photo-thermal efficacy of dyes is not hindered at all by the encapsulation of dyes into liposomes or EMVs.

Finally, dye stability after laser irradiation was evaluated by comparing absorbance peak values before and after irradiation. The calculated absorbance decrease (%) for all samples is shown in Figure 9d. Laser power again proved to have an influence on free ICG in PBS, with a lower degradation rate at P = 0.5 W/cm^2^ (34.8%) than at P = 3 W/cm^2^ (73.6%). These results (ICG 10 μg/mL) coincide well with ICG solutions in PBS tested previously (Table 1, ICG concentrations 5, 25, and 50 μg/mL). Interestingly, ICG nano-carriers degraded similarly (around 50% for 3 W/cm^2^ and 25% at 0.5 W/cm^2^) at both laser powers. Such a decrease in peak absorbance (measured at 800 nm, i.e., peak for the monomeric form) was remarkably lower in the carrier rather than as free ICG (significant, with significance set at *p* > 0.05, according to Brown–Forsythe corrected ANOVA) in the case of both 0.5 and 3 W/cm^2^. This behavior can be explained by the stabilization effect of the monomeric form of ICG due to interaction with lipids and proteins; degradation is slowed down since the dye does not form H or J aggregates; therefore, diffusion of singlet oxygen, mainly responsible of ICG degradation, is reduced, too [8]. In particular, singlet oxygen diffusion in carriers can be decreased by the presence of PEG2000 in liposomes and unsaturated fatty acids and proteins in EMVs [31,32]. Such phenomenon has been already observed for dextran mesocapsules [33], in PEG-poly(ε-caprolactone) micelles [34], and in DSPE-PEG micelles as well [35]. In addition, all IR-820 samples had a similar degradation profile, no matter whether in free or encapsulated form (52% at P = 0.5 W/cm^2^ and 68% at P = 3 W/cm^2^), again showing that in the case of IR820, encapsulation does not protect the dye from photo-degradation. While for ICG, localization and degradation mechanisms are well known, the same cannot be said for IR820, as already proven that embedding within a carrier (PGLA) does not improve photostability [36]. However, as mentioned before, encapsulation in nano-carriers can offer a prolonged circulation time at the same photo-thermal efficacy (same achieved temperature increase as in the case of free dyes).

### 2.7. Hemolysis Study

**Figure 10:**
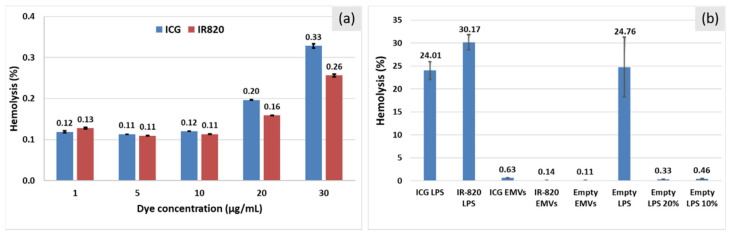
Influence of ICG and IR-820 dyes on red blood cells toxicity. All measurements were performed in triplicate. (**a**) Hemolysis determination of PBS solutions of dyes in the concentration range from 1 to 30 μg/mL after 3 h incubation at 37 °C. Data analysis revealed no statistically significant difference in the cell viability values between tested groups (ICG and IR-820 at a given concentration, *p* > 0.05) up to 10 μg/mL. At a higher concentration (20 and 30 μg/mL), hemolysis values become significantly different between tested groups (*p* < 0.05). (**b**) Hemolysis determination of dye-containing liposomes (LPS) and EMVs after 3 h incubation at 37 °C. Empty carriers were tested as control groups. Liposomal samples (without dilution) showed a statistically significant difference between hemolysis values (ANOVA test, F > F critical = 20.09 > 5.14, *p* < 0.005). Hemolysis values for all EMV samples differed significantly (ANOVA test, F > F critical = 34.89 > 5.14, *p* < 0.001).

To evaluate the possible hemotoxicity of dyes, ICG and IR-820 solutions in PBS (1–30 μg/mL) were incubated with red blood cells (RBCs, 5 vol.%) for 3 h (Figure 10a). Hemolysis was found negligible (0.1%) up to 10 μg/mL for both dyes, with a slight increase at the highest tested concentrations (0.33% and 0.26% at 30 μg/mL of ICG and IR-820, respectively). Then, the hemolysis after incubation with dye-loaded nano-carriers was evaluated (Figure 9b, dye concentration 10 μg/mL). All EMV-based samples showed minimal hemolysis, while, strikingly, liposomes induced severe hemolysis. Since this was observed for empty liposomes, too, we further investigated the origin of the hemotoxicity. Reducing the liposome concentration by 5 and 10 times resulted in drastic hemolysis reduction (Figure 10), indicating hemotoxicity as a consequence of high phospholipid concentration (5 mM in initial samples). This formulation is clinically approved (DPPC/MSPC/DSPE-mPEG2000 = 90:10:4 molar ratio, i.e., low-temperature-sensitive formulation, ThermoDox^®^; Celsion Corporation, USA). However, it apparently induces hemotoxicity, which could be originating from the lysolipid component (MSPC). It was demonstrated [37] that under ideal conditions (absence of plasma proteins, other double layers than liposomes), the lysolipid is kept in the lipid double layer below the phase transition temperature (approx. 41 °C for this formulation). Instead, under real-life conditions, MSPC can be released from the liposomes and fuse with other bilayers [38], such as RBCs in our case. It was observed [37] that after injection in mice, the concentration of marked MSPC kept on decreasing in hematic flux. Unfortunately, hemotoxicity was not assessed. The authors, instead, incubated the Thermodox formulation with whole blood, discovering that over 4 h, a small but important loss of lysolipid occurs, enough to dramatically reduce the concentration of the drug within the liposomes, which indicates a disturbance in both double layers. On the contrary, we assayed in a quantitative way only from the erythrocytes’ point of view. To confirm this assumption, we then tested formulations without MSPC (DPPC:DSPE-mPEG2000) and observed a drastic decrease in hemolysis down to 8.1% at 5 mM (compared to 37.3 % for the full formulation). Further dilution (5 and 10 times) resulted in the measured hemolysis at a level of control samples (<0.1%). In conclusion, from the hemolytic point of view, EMVs prove to be less hemotoxic dye carriers than clinically approved lipid formulations.

## 3. Materials and Methods

### 3.1. Chemicals

Indocyanine green (ICG, laser grade) was purchased from Acros Organic (a Thermo Fisher Scientific company, Morris Plains, NJ, USA), and IR820 (dye content 80%) and CHCl_3_ (>99%) were purchased form Sigma-Aldrich (Sigma-Aldrich, Munich, Germany ). Liposomes were prepared using 1,2-dipalmitoyl-sn-glycero-3-phosphatidylcholine (DPPC), 1-myristoyl-2-stearoyl-sn-glycero-3-phosphocholine (MSPC), and N-[carbonyl-methoxy(polyethylene glycol)-2000]-1,2-distearoyl-sn-glycero-3-phosphoethanolamine, a sodium salt (DSPE-PEG2000) purchased from Lipoid GmbH (Ludwigshafen, Germany). For EMV preparation, the following chemicals were used: phosphate-buffered saline buffer (PBS, tablets; Sigma Aldrich), Alsever’s medium (TCS Biosciences Ltd., UK), and NaCl (Sigma-Aldrich, >99.5%).

### 3.2. Preparation of Dye-Loaded Liposomes

Liposomes were prepared by a thin-film hydration method followed by extrusion [39]. Briefly, 8.6 µmol of DPPC (10 mg/mL), 1 µmol of MSPC (5 mg/mL), and 0.4 µmol of DSPE-PEG2000 (10 mg/mL) were dissolved in CHCl_3_. The organic mixture was transferred to a round-bottom flask (25 mL), and then CHCl_3_ was removed at 40 °C using a rotary evaporator (Rotavapor R-100 equipped with a heating bath, Interface I-100, and vacuum pump V-100; Bunchi, Switzerland). Once a lipid film was formed, the pressure in the flask was reduced to 10 mbar for 1 h. The dried lipid film was then hydrated with 2 mL of 30 μg/mL of ICG or IR-820 dye in 0.1× PBS in a circulating flask at 60 °C for 30 min. Particle size was reduced using a mini-extruder (Avanti Polar lipid, USA). Samples were extruded at 60 °C through 800 nm (5 cycles), 200 nm (15 cycles) and lastly through 100 nm membranes (25 times, PC membrane filters; Avanti Polar lipid, USA), left to anneal for 2 h at room temperature and stored at 4 °C for further experiments.

### 3.3. Preparation of Dye-Loaded EMVs

Whole blood of sheep was supplied by the Veterinary Faculty (University of Ljubljana, Slovenia) in Alsever’s medium (pH 6.1) and used within 1 week. Erythrocytes were isolated via centrifugation (2500 rpm/10 min) and washed 3 times with PBS buffer. Plasma was used for stability study purposes. For the preparation of empty EMVs, 3 mL of 20 vol.% of erythrocytes in PBS buffer was centrifuged (model 5804 Eppendorf) at 8000 rpm for 10 min. The supernatant was discarded, and the pellet was redispersed with 0.1× PBS, incubated for 20 min at 4 °C to release the hemoglobin and centrifuged at 8000 rpm. The supernatant was discarded. The incubation–centrifugation step was repeated until the supernatant was clear and only a white pellet of cell membranes remained. As prepared, empty EMVs were then redispersed in 1 mL of 30 μg/mL dye in 1× PBS and incubated at room temperature for 15 min. Afterward, the samples were homogenized using an ultrasound finger (ultrasonic processor Sonics Vibra cell VC-505; pulses: 1 s on, 1 s off; 20% amplitude). To reduce the particle size, samples were extruded at 37 °C through 800 nm (5 cycles) and 200 nm (15 cycles) membrane filters. EMVs were left to anneal for 2 h at room temperature and stored at 4 °C for further experiments.

### 3.4. Purification of Dye-Loaded EMVs/Liposomes and Encapsulation Efficiency

The purification step involved removal of the non-encapsulated dye from liposomes and EMVs by gel filtration using a PD-10 column (GE Healthcare, UK) equilibrated with 1× PBS. Briefly, 1 mL of the extruded sample was inserted into the column, and seven fractions (1 mL each) were collected. The presence of liposomes/EMVs was proved via particle size and absorbance measurements of each fraction. Only in fractions 3 and 4, liposomes with a size of around 115 nm and a considerable count rate were detected, which was further supported with absorbance measurements using 96-well transparent F-bottom microplates and a Synergy H4 Hybrid microplate reader (BioTek, Winooski, VT, USA). A 2× dilution factor was considered when calculating the encapsulation efficiency (EE%). The same procedure was applied for EMVs. Fractions 4 and 5 were collected, having a considerable count rate and a particle size of 220–230 nm on average.

### 3.5. Hydrodynamic Diameter and Zeta Potential Measurements

The hydrodynamic diameter, polydispersity index (PDI), and zeta potential (ζ-potential) of empty and dye-containing liposomes and EMVs were determined using a zeta potential and particle size analyzer ZetaPALS (Brookhaven Instruments Corporation, New York, NY, USA) at 25 °C. Disposable polystyrene cells (Q-Vettes; Ratiolab, Germany) were used for particle size and zeta potential measurement.

### 3.6. Absorption Spectra of IR-820 and ICG Solutions

Due to the limited solubility of dyes in phosphate-buffered saline (PBS), 0.9% saline, and blood plasma, ICG and IR-820 were first dissolved in ultrapure water to obtain a dye concentration pf 0.5 mg/mL and then diluted further with PBS, 0.9% saline, or blood plasma to reach working solutions in the concentration range of 1–50 μg/mL. Measurements were performed using a Lambda950 UV/VIS/NIR spectrometer (PerkinElmer, Waltham, MA, USA), quartz cuvettes, and a microplate reader with 96-well plates. Absorption spectra were recorded in the wavelength range from 500 to 900 nm.

### 3.7. Temperature Stability of IR-820 and ICG Solutions

ICG and IR-820 solutions (10 μg/mL) in water, PBS, 0.9% NaCl(aq), and blood plasma were incubated for 24 h at 4, 25, and 37 °C using a microplate reader. Absorbance peak values were recorded every hour. Final and initial values were used for calculation of the percentage absorbance decrease, which served as an indicator of dye degradation. Similarly, temperature stability of ICG- and IR-820-loaded liposomes and EMVs (all with dye concentration 10 μg/mL) was determined via 24 h incubation at 25 and 37 °C using a microplate reader, following the above-described protocol. A concentration of 10 μg/mL was chosen since it is diluted enough to have monomeric forms of ICG and IR820 to be predominant. The reaction volume was 1 mL, and the thermal expansion of water was negligible. All samples were allowed to cool at room temperature before final spectra measurement, the latter being used as an indicator of dye degradation, to avoid water thermal expansion concentration bias.

One-way ANOVA and unpaired Student’s *t*-test was used for our statistical analysis, assuming normal distribution of data. The differences were considered significant at *p* < 0.05. Data were shown as the mean ± SD (*n* = 3).

### 3.8. Photo-Stability of IR-820 and ICG Solutions

Photo-thermal experiments were performed using an FC-808 Fiber Coupled Laser System (CNI Optoelectronics Tech, Changchun, China) configured for continuous-wave operation at 808 nm with different powers. Laser light was focused on a quartz cuvette (size 1 × 1 × 3 cm^3^) using an optical lens with a spot size of about 8 mm. Control (PBS buffer) and dye-containing solutions were irradiated at laser power P = 0.5, 1, and 3 W/cm^2^ for 5 min. Such duration was chosen to mimic an in vivo photo-thermal experiment [7,40]. The temperature of the liquid samples (volume 1 mL) was measured with a J-type Teflon thermocouple that was immersed in the cuvette and connected to a computer to collect the data in real time. To evaluate photo-induced degradation, absorbance spectra in the range of 500–900 nm were recorded before and after laser irradiation. In the case of absorbance spectra recorded after irradiation, the impact of the degradation products of dyes, influencing the ionic strength of samples, was assumed to be negligible and to not influence the shape and reliability of the remaining ICG molecules’ spectrum [8]. Loaded carriers were prepared, as described in Section 3.2 and Section 3.3, avoiding the extrusion process and only sonicating the samples in order to keep the initial concentration of the dye constant and to ensure no experimental bias due to differential encapsulation efficiency. Brown–Forsythe ANOVA was employed to evaluate the consistency of observed data. Differences were considered relevant at *p* < 0.05. Data were shown as the mean ± SD (*n* = 3).

### 3.9. Hemotoxicity Study

To assert the hemolytic activity of liposomes and EMVs, a hemoglobin leakage assay was used [41]. Briefly, erythrocytes were isolated from the whole blood of sheep, as described in the section for the preparation of EMVs. Control (PBS only), dyes in PBS (1–50 μg/mL), empty liposomes, empty EMVs, and dye-loaded liposomes and EMVs were incubated with 5 vol.% of erythrocytes in PBS (pH 7.4) for 3 h at 37 °C with constant orbital shaking in 1.5 mL tubes (Eppendorf, Germany; volume of samples 1 mL; all samples in triplicate). After incubation, tubes were centrifuged (1500 rpm/4 min) to sediment cells, and the supernatant was analyzed in triplicate. Hemolysis was evaluated by measuring the released hemoglobin absorbance (*A*) at 541 nm using a plate reader. Samples representing “100% dead” were prepared by lysing control samples with deionized water via hypotonic osmotic shock. The percentage hemolysis was then calculated as follows:

(1) $$\mathrm{Hemolysis}\;(\%)=100\times \frac{\left(A_{\mathrm{sample}}-A_{\mathrm{control}}\right)}{\left(A_{100\%\;\mathrm{dead}}-A_{\mathrm{control}}\right)}$$

One-way ANOVA and Student’s *t*-test were used for our statistical analysis. Data were presented as the mean ± SD for all experiments.

## 4. Conclusions

This study provided enough experimental evidence to answer the burning questions that were raised in the Introduction section. First, we found that encapsulation of dyes in liposomes and EMVs can improve the dyes’ thermal stability. In addition, specific carrier encapsulation can prevent quick photo-degradation, due to stabilization of the monomeric form, in the case of ICG, known to locate within double layers; a carrier then has to be carefully engineered to reduce the singlet oxygen diffusion rate. Second, IR-820 exhibits similar stability but a lower photo-thermal conversion efficacy than ICG at the same dye concentration and is thus a less appropriate photo-thermal agent than ICG, and carriers are not proven to be able to protect it from photo-degradation, giving hints of its localization within them. More studies are needed to better characterize IR820 localization and interaction with biological matter. Finally, DPPC/DSPE-PEG2000/MSPC liposomes themselves were found to be hemotoxic at as high concentrations as 5 mM, which highlight the superiority of EMVs regarding the hemocompatibility profile. In conclusion, the use of the body’s own cells as nano-carriers for dyes and other active components is a highly attractive strategy that might help overcome the challenges current nanomedicine is facing.

## Figures and Tables

**Table 1:** Calculated absorbance decrease after irradiation with 808 nm laser (P = 0.5, 1, and 3 W/cm^2^) of ICG solutions in PBS. An increase in temperature (*ΔT*) was calculated by subtracting the initial temperature (*t* = 0 s) from the maximum temperature reached.

Concentration (μg/mL)	P (W/cm^2^)	Absorbance before Irradiation	Absorbance after Irradiation	% Degradation	*ΔT* (°C)
5	0.5	0.748	0.295	60.5	3.8
5	1.0	0.748	0.237	68.3	5.1
5	3.0	0.748	0.145	80.6	7.5
25	0.5	2.326	1.884	19.0	13.2
25	1.0	2.326	1.398	39.9	21.8
25	3.0	2.326	0.375	83.9	31.1
50	0.5	3.678	3.567	3.0	12.8
50	1.0	3.678	3.197	13.1	24.2
50	3.0	3.678	1.182	67.9	50.7

**Table 2:** Calculated absorbance decrease after irradiation with 808 nm laser (P = 0.5, 1, and 3 W/cm^2^) of IR-820 solutions in PBS. An increase in temperature (*ΔT*) was calculated by subtracting the initial temperature (*t* = 0 s) from the maximum temperature reached.

Concentration (μg/mL)	P (W/cm^2^)	Absorbance before Irradiation	Absorbance after Irradiation	% Degradation	*ΔT* (°C)
5	0.5	0.160	0.070	56.3	1.8
5	1.0	0.160	0.056	64.8	2.0
5	3.0	0.160	0.036	77.4	3.4
25	0.5	0.491	0.150	69.5	4.8
25	1.0	0.491	0.117	76.1	10.0
25	3.0	0.491	0.095	80.7	8.0
50	0.5	1.410	0.655	53.5	11.7
50	1.0	1.410	0.271	80.8	19.0
50	3.0	1.410	0.093	93.4	27.3

**Table 3:** Particle size, polydispersity index (PDI), and dye encapsulation efficacy (EE) of empty, ICG-, and IR-820-loaded liposomes and EMVs (*n* = 3).

Sample	Size (nm)	PDI	EE (%)
Empty LPS	115 ± 1	0.087 ± 0.050	-
Empty EMVs	234 ± 4	0.238 ± 0.011	-
ICG LPS	126 ± 3	0.171 ± 0.083	45 ± 3
ICG-EMVs	215 ± 5	0.198 ± 0.075	57 ± 2
IR-820 LPS	116 ± 2	0.142 ± 0.091	46 ± 4
IR-820 EMVs	260 ± 4	0.229 ± 0.081	53 ± 3

## Data Availability

Not applicable.
